# Experimental measurement-device-independent quantum digital signatures

**DOI:** 10.1038/s41467-017-01245-5

**Published:** 2017-10-23

**Authors:** G. L. Roberts, M. Lucamarini, Z. L. Yuan, J. F. Dynes, L. C. Comandar, A. W. Sharpe, A. J. Shields, M. Curty, I. V. Puthoor, E. Andersson

**Affiliations:** 1 Toshiba Research Europe Ltd, 208 Cambridge Science Park, Cambridge, CB4 0GZ UK; 2 Cambridge University Engineering Department, Cambridge, CB3 0FA UK; 3 EI Telecomunicación, Department of Signal Theory and Communications, University of Vigo, Vigo, E-36310 Spain; 4 SUPA, Institute of Photonics and Quantum Sciences, Heriot-Watt University, Edinburgh, EH14 4AS UK

## Abstract

The development of quantum networks will be paramount towards practical and secure telecommunications. These networks will need to sign and distribute information between many parties with information-theoretic security, requiring both quantum digital signatures (QDS) and quantum key distribution (QKD). Here, we introduce and experimentally realise a quantum network architecture, where the nodes are fully connected using a minimum amount of physical links. The central node of the network can act either as a totally untrusted relay, connecting the end users via the recently introduced measurement-device-independent (MDI)-QKD, or as a trusted recipient directly communicating with the end users via QKD. Using this network, we perform a proof-of-principle demonstration of QDS mediated by MDI-QKD. For that, we devised an efficient protocol to distil multiple signatures from the same block of data, thus reducing the statistical fluctuations in the sample and greatly enhancing the final QDS rate in the finite-size scenario.

## Abstract (web summary)

Measurement-device-independent quantum digital signatures would allow a document to be signed and transferred with information-theoretic security. Here, the authors reach this goal using a reconfigurable quantum network where the central node can switch between trusted and untrusted operation.

## Introduction

Cryptography is ubiquitous in modern society and essential to countless applications relying on the confidentiality, integrity and non-repudiation of sensible data^1^. Currently, the security of these applications is largely based on public-key cryptography^2, 3^, which is supposedly secure against an eavesdropper with limited computational power. Quantum key distribution (QKD), on the other hand, poses no restrictions on the attacker, apart from obeying the laws of nature, and one only makes assumptions on the devices owned by the authorised users, which can be directly tested^4^. In addition, the recent introduction of measurement device independent (MDI)-QKD^5, 6^ further enhances the positive features of QKD. By clever use of the teleportation gate^7^, MDI-QKD turns the receiving side of QKD into a transmitter, thus removing all the security assumptions on the detecting devices, which are arguably most exposed to external attacks^8–12^. Moreover, it allows two parties to connect through a totally untrusted node, which is particularly important in a network configuration^13^. Recent experiments have shown that it can be implemented with key rates commensurate to those of QKD^14^, while extending its transmission distance^15^.

Different from encryption, digital signatures play a vital role in software distribution, modern communication and financial transactions, where the integrity of the data against forgery is of utmost importance. While they are currently implemented using public-key cryptography, quantum digital signatures (QDS) have recently been introduced^16–18^ to allow users to sign a document by quantum means and transfer it to other users with information-theoretical security. Quantum signatures were introduced in ref. ^16^, but that scheme was impractical as it required the use of a quantum memory. Recent developments have removed this limitation^17, 18^ and made the QDS techniques closer to those employed in QKD^19^ and MDI-QKD^20^. The resulting schemes, however, have limitations. The one in ref. ^19^ needs essentially one QKD link for each pair of users taking part in the distribution of QDS. In a network with *N* users, this would amount to *N*(*N* − 1)/2 direct physical links, an impractically high number for a large number of users. In the configuration described in ref. ^20^, on the other hand, a central node connects pairs of users via MDI-QKD, but cannot directly communicate with them. So a simple yet crucial operation like a digitally signed firmware update from the central node to the end users would be impossible with this scheme.

Here, we propose and experimentally realise a quantum network concept to overcome all of the above limitations. The network can connect three users by means of only two optical links, entailing a favourable scaling of *N* − 1 links for an *N*-node network. This is achieved by configuring the central node as an entirely untrusted relay that connects the end users via MDI-QKD. However, the central node can also be reconfigured so to act as a trusted recipient and communicate directly and securely with the end users via QKD. Because the network is fully connected, we could use it to distil quantum encryption keys and quantum digital signatures between all pairs of users. In particular, this allows us to extract the first QDS rates mediated by MDI-QKD. Using newly adapted finite-size distillation protocols (all details are given in Supplementary Note 1), we obtain key rates around 10^4^ bits per second (bps) for MDI-QKD on a 50-km optical fibre and 10^6^ bps for QKD on a 25-km optical fibre, as well as an MDI-QKD-mediated QDS rate of 1 signed bit every 45 s and a QKD-mediated QDS rate of 1 signed bit every 72 ms. This performance would enable high-speed applications in future quantum networks.

## Results

### System schematics

**Fig. 1:**
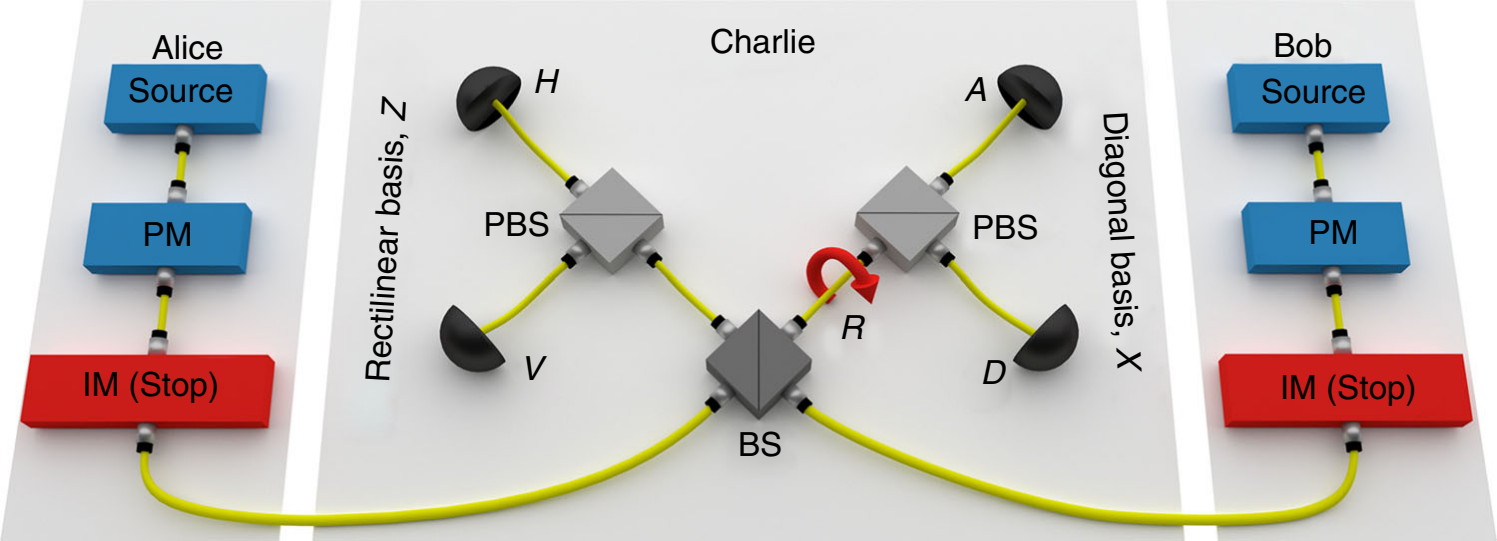
Fibre-based MDI/QKD network. The key elements are coloured red. The rotator (R) in Charlie’s station sets each detector to measure a different polarisation state, *H* horizontal, *V* vertical, *D* diagonal and *A* anti-diagonal. The intensity modulators (IMs) can be set to high attenuation, to nearly stop the light passing through them (“Stop” in the figure). This can enable Alice-Bob MDI-QKD, when no IM is set to Stop, or Alice-Charlie QKD, when Bob’s IM is set to Stop, or Bob-Charlie QKD, when Alice’s IM is set to Stop. At the same time, the IMs can be used to prepare decoy states^21–24^. PM, polarisation modulation; BS, beam splitter; PBS, polarising beam splitter

The schematics of the network are shown in Fig. 1. Two distant users, Alice and Bob, are connected through a central node, Charlie, who normally acts as an untrusted relay between the users. In this case, the confidentiality of the communication between Alice and Bob is guaranteed by the fact that they use the two intensity modulators (IM) to run the decoy-state^21–24^ MDI-QKD protocol^6^. The resulting key will then be unknown to Charlie and to any external eavesdropper, and secure against attacks directed at Charlie’s equipment. To let Alice and Bob communicate directly with Charlie, point-to-point QKD links can be activated, as explained shortly. If the users’ devices are trusted, the transmission in the QKD modality will feature quantum security against external eavesdroppers and will be fast, with megabits of key material distributed every second. In the scheme in Fig. 1, a QKD transmission between Alice (Bob) and Charlie is enabled by stopping the light emitted by Bob (Alice) through the same IMs employed for the decoy-state MDI-QKD protocol. Hence, the IMs are key components in the setup, allowing switching between QKD and MDI-QKD and, simultaneously, the implementation of the decoy-state technique. The possibility to switch between QKD and MDI-QKD constitutes a “reconfigurable MDI/QKD network”, a concept similar to the one introduced in ref. ^25^ for free-space quantum communications.

### Experimental setup

To implement the network in Fig. 1, we adopt a polarisation-based setup. We denote by *H*, *V*, *D* and *A* the horizontal, vertical, diagonal and anti-diagonal states of linear polarisation, respectively, and with *Z* and *X* the rectilinear and diagonal bases, composed of the states {*H*, *V*} and {*D*, *A*}, respectively. The setup makes use of the decoy-state technique to improve the key rate and extend the transmission distance. Therefore, the preparation step also includes the selection of the intensity of the pulses to be sent to Charlie. In this case, we adopt the scheme with four intensity classes^14, 26^, indicated as *s* (“signal”), *u* (“decoy1”), *v* (“decoy2”) and *w* (“vacuum”). The signal *s* is the only one prepared in the *Z* basis, whereas *u*, *v* and *w* are all prepared in the *X* basis. The quantum keys and signatures are extracted from the *s* pulses in the *Z* basis, whereas the *X* basis is for testing the quantum channel against the presence of an eavesdropper. To increase the final key rate, the basis *Z* is selected more often than *X*.

**Fig. 2:**
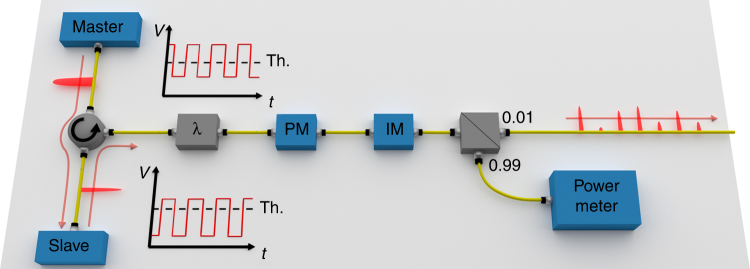
Transmitting module. Two replicas of the depicted fibre-based setup are used by Alice and Bob to transmit light pulses to Charlie. Master and slave lasers’ driving signals are displayed alongside the equipment and are set differently for the two lasers. A power metre connected to the output beam splitter attenuates the outgoing optical pulses to the correct level. The wavelength filter (λ) is for enhancing the indistinguishability of Alice’s and Bob’s photons

The preparation of the pulses in the experimental setup is effected through the transmitter depicted in Fig. 2. Alice and Bob create low-jitter 32-ps light pulses at 1549.8 nm using the pulsed laser seeding technique^14^. The master laser is input to the slave via a circulator and the AC voltage is temporally offset between the two lasers to ensure injection occurs at the correct time. The 1-GHz gain switching of both lasers ensures that all pulses are phase randomised^27, 28^. Alice and Bob’s pulses are then passed through separate 30 GHz bandwidth filters to remove noise. The polarisation of the pulses is controlled using electric polarisation controllers, which can create all of the required polarisation states. An attenuator provides the four photon fluxes (*s*, *u*, *v*, *w*) before they are sent to Charlie.

Charlie is composed of the interfering beam splitter (BS), two polarising BS (PBS) aligned along the *Z* axis, one polarisation rotator (R) and four InGaAs self-differencing avalanche photodiodes, run at room temperature, clocked at 1 GHz and featuring an average efficiency of 20.9%. The rotator R placed after one output port of Charlie’s BS turns a *Z*-basis analyser into an *X*-basis analyser. This is important for enabling reconfigurable MDI/QKD as it allows the realisation of a full QKD receiver, measuring the incoming pulses in two complementary bases. On the other hand, all the coincidence counts from detectors *H* and *V* can be treated as in the original MDI-QKD scheme, whereas coincidence counts from detectors on different output ports of Charlie’s BS cannot be used to distil key bits, as they belong to different bases. A coincidence count between *H* and *V* indicates projection onto the triplet Bell state $\left| {{\psi ^ + }} \right\rangle = 1{\rm{/}}\sqrt 2 \left( {\left| {HV} \right\rangle + \left| {VH} \right\rangle } \right) = 1{\rm{/}}\sqrt 2 \left( {\left| {DD} \right\rangle - \left| {AA} \right\rangle } \right)$. In this case, Bob flips (does not flip) his bit to match Alice’s bit if the rectilinear (diagonal) basis was used in the preparation step. The same argument applies to the *X*-basis branch, by replacing *H* with *D* and *V* with *A*. Alice, Bob and Charlie share a common reference clock, allowing Alice and Bob to align their pulses, so to arrive coincidentally at Charlie, and allowing Charlie to align his detectors.

To effect the selection between MDI-QKD and QKD, Alice and Bob act on their IMs to send or stop the light directed to Charlie. In particular, when Alice (Bob) prepares the vacuum state *w*, the amount of light travelling towards Charlie is so small that the situation is virtually identical to having the AC (BC) link disconnected and QKD enabled on the BC (AC) link. Any potential residual light in the vacuum state does not affect the security of the scheme, as it directly translates into an increase of the measured quantum bit error rate (QBER). Also, if there are multiple counts in Charlie’s detectors during the QKD sessions, they can be treated using the squashing model for the passive BB84 protocol^29, 30^. Finally, in some cases, neither Alice nor Bob will prepare a vacuum state, whereas in other cases they both will. Such instances can be employed to enable an MDI-QKD communication (see details in Supplementary Note 1).

### Key rates for encryption

**Fig. 3:**
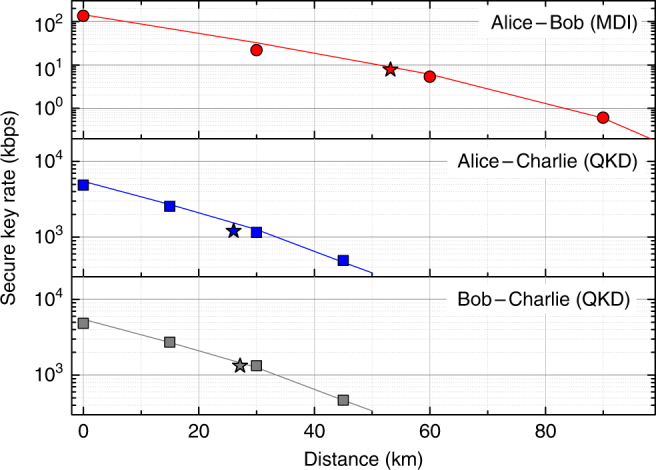
Secure key rates vs. distance. MDI-QKD (top diagram) and QKD (centre and bottom diagrams) secure key rates for a *Z* (*X*) basis probability equal to 80% (20%) and a security parameter ${\epsilon _{{\rm{sec}}}}$ ≲ 10^−10^ are shown as a function of distance. Because the reconfigurable scheme is symmetrically deployed, the distance between Alice and Bob (top) is approximately twice the distance between Alice and Charlie (centre) and Bob and Charlie (bottom). All the distances have been calculated assuming 0.2 dB/km attenuation on the channel, except for the data points indicated by stars, where a real fibre was used. The projected time to attain the shown key rates is 25 h of which 3 min are spent on QKD and the rest on MDI-QKD, to balance their key rates. The fitting lines are the result of a numerical simulation with the parameters mentioned above and the following additional ones: Charlie’s insertion loss, 1.1 dB; detectors’ efficiency and temperature, 20% and 0°, respectively; afterpulsing, 4%; dark count probability per gate, 1.6 × 10^−5^; error correction coefficient, 1.16

Using the described setup, we run QKD and MDI-QKD experiments, deriving key rates vs. distance, as depicted in Fig. 3. We performed two sets of experiments. In the first, we used variable optical attenuators to simulate a lossy channel with 0.2 dB/km, as in a typical optical fibre at 1550 nm. In the second, we used two 25-km reels of a standard optical fibre. The circles (squares) are for the attenuator-based MDI-QKD link (QKD links), whereas the stars represent the points obtained using a real fibre. Finally, the solid lines are theoretical simulations tailored to our experimental setup. Tables containing all the measured counts are reported in Supplementary Tables.

From Fig. 3, it is apparent that the theory reproduces the experimental results well, both for attenuators and real fibre, with only a slightly lower experimental rate for the fibre due to a correspondingly higher QBER. This allowed us to use a simulation to optimise the system before performing real experiments. The key rates for MDI-QKD are between 606 bps for an equivalent distance of 90 km and 134 kbps for 0 km. QKD is faster, providing key rates ranging from about 0.5 Mbps at 45 km to almost 5 Mbps at 0 km. This difference in the key rates led us to set the probability of an MDI-QKD run equal to 500 times that of a QKD run. In a network with three users, this would provide comparable key rates for all users, on average, over all distances. Before performing the experiment, the setup was optimised for MDI-QKD. Then, key rates were acquired for both MDI-QKD and QKD without additional calibrations.

Key rates in Fig. 3 were calculated using composable security proofs in the finite-size scenario^31, 32^ with a procedure similar to the one described in ref. ^14^. With the proviso that the key bits are extracted in the *Z* basis, we drop the index *Z* and write them as:

1 $${R^{{\rm{MDI}}}} = {\underline S ^{1,1}}\left[ {1 - h\left( {\overline e _{{\rm{ph}}}^{1,1}} \right)} \right] - {\rm{leak}}_{{\rm{EC}}}^{{\rm{MDI}}} - {{\rm{\Delta }}^{{\rm{MDI}}}},$$

2 $${R^{{\rm{QKD}}}} = {\underline S ^1}\left[ {1 - h\left( {\overline e _{{\rm{ph}}}^1} \right)} \right] - {\rm{leak}}_{{\rm{EC}}}^{{\rm{QKD}}} - {{\rm{\Delta }}^{{\rm{QKD}}}}.$$

In Eqs. (1) and (2), the labels “MDI” and “QKD” refer to MDI-QKD and QKD, respectively. The quantities *S* and *e*_ph_ indicate single-photon counts and single-photon phase-error rate, respectively, in the *Z* basis and intensity class *s*, estimated by applying the decoy-state technique to the *X*-basis data sample and then extending to the *Z* basis using standard statistical tools (see Supplementary Note 1). The function *h* is the binary entropy. The upper and lower bars are for upper and lower bounds and the superscripts “1” or “1,1” refer to one sender (QKD) or two senders (MDI-QKD) emitting single photons. The quantity leak_EC_ is the amount of bits used to correct errors in the *Z* basis, while the Δ terms take into account the finite-size effect.

### Quantum digital signatures

Having demonstrated the capability to distil encryption keys between all the nodes in the network, we now turn on QDS and describe specifically our method to extract QDS rates from an MDI-QKD link. This is not a trivial extension of the previous cases because, differently from encryption, the goal of digital signatures is demonstrating the authenticity of a signed message to multiple recipients rather than keeping it secret. So the quantum protocols devised for encryption keys have to be adapted to QDS (see Supplementary Note 1).

So far, various proof-of-concept experiments have been performed on QDS^33–35^, with the most recent ones reaching distances up to 90 km in optical fibre^35^. However, this was achieved using a protocol secure only against individual attacks in the asymptotic scenario. Moreover, previous schemes were either realised over very short distances^33, 34^ or on a single optical fibre to represent two of the three necessary links for QDS^35^. Finally, no previous experiment has used MDI-QKD to implement QDS.

**Fig. 4:**
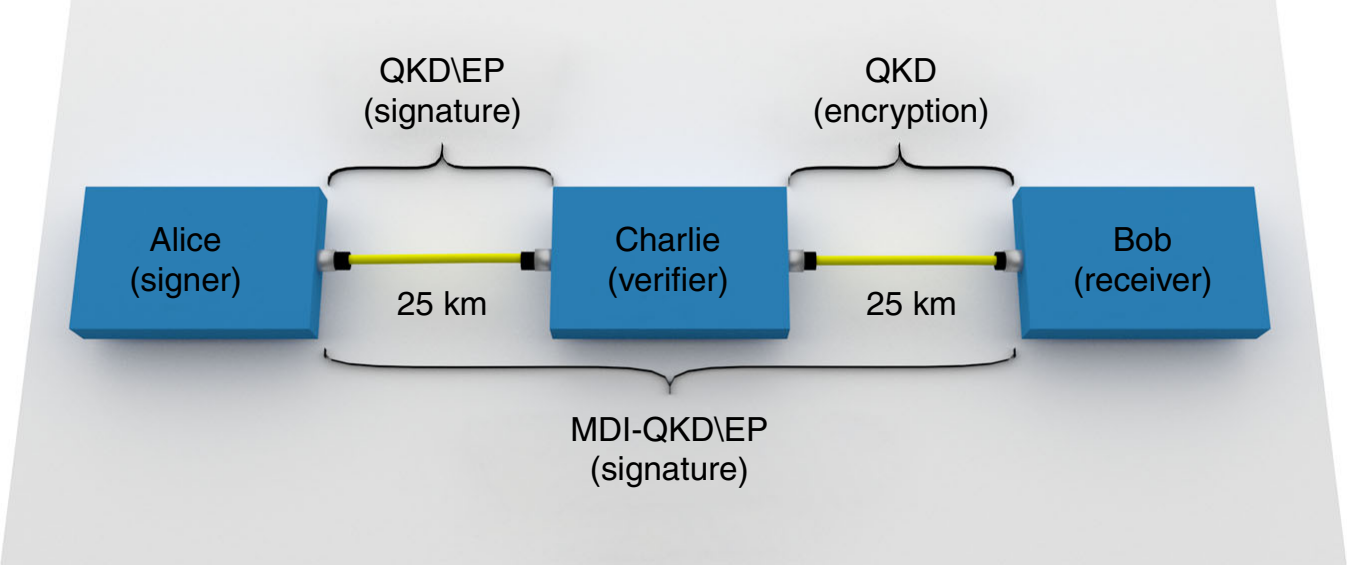
MDI-QKD-mediated QDS. The signature is sent by Alice to Bob using an MDI-QKD setup, over an optical fibre with a total length of 50 km. The protocol is denoted “MDI-QKD\EP”, where “\EP” stands for “without error correction and privacy amplification”. QKD\EP is used to send a signature from Alice to Charlie, whereas full QKD is used to distribute keys between Bob and Charlie to allow for the symmetrisation step of QDS (see Supplementary Note 1). The QKD links are implemented with two 25-km reels of single-mode fibre

In the simplest case, a QDS scheme involves three parties, as depicted in Fig. 4. One of them, Alice, signs a document and sends it to a receiver, Bob, who accepts it after checking that the signature is genuine. The same document can also be transferred to a third user, Charlie, for verification purposes. We implement QDS using the quantum network in Fig. 1. Two 25-km optical fibres connect Alice and Bob to Charlie, whereas there is no direct fibre between Alice and Bob, who are linked only by the intermediate node using MDI-QKD. We choose this particular realisation of QDS to demonstrate signature distribution based on MDI-QKD. However, given the key rates represented by stars in Fig. 3, we could also have used the setup with the signing party at the “Charlie” node, in which case MDI-QKD would be used to encrypt the symmetrising exchange of signature bits between the two recipients at the “Alice” and “Bob” nodes. Therefore, our demonstration is not limited to the particular case represented in Fig. 4. Let us also remark that, to strengthen our demonstration, we used real fibre to perform the QDS experiment.

To demonstrate QDS mediated by MDI-QKD, we adopt MDI-QKD without error correction and privacy amplification (denoted “MDI-QKD\EP”) for the signature between Alice and Bob, and QKD without error correction and privacy amplification (QKD\EP) for the signature between Alice and Charlie. Full QKD between Bob and Charlie is also used, to enable the symmetrisation step of the protocol (see Supplementary Note 1 for details). All the results in the *X* basis are publicly revealed, whereas only a small portion of the *Z* basis results is disclosed, to estimate the QBER in this basis. The remaining undisclosed bits are used for quantum digital signatures.

### Quantum digital signature rates

Here, we extract the specific parameters of a QDS protocol, i.e., the size of signatures and the security parameters against forging and non-repudiation^19, 20^. For that, we introduce a protocol with a refined finite-size analysis, to increase the QDS rate. In the finite-size scenario, the users acquire data for a finite amount of time, until the data block is large enough to guarantee small statistical fluctuations in the parameters estimated from the data set. Then they proceed and acquire the next block of data. The current approach for QDS is to distil a single signature from every block of data. Therefore, to increase the QDS rate, it is optimal to keep the data block as small as possible, so as to have more blocks in a given time interval. This, however, is highly inefficient. It makes statistical fluctuations larger, thus worsening the estimation of the quantum-related parameters and the QDS rate.

In our protocol, we still perform a single decoy-state parameter estimation per each block of data, but we extract multiple signatures from the same data block (see details in Supplementary Note 1). This allows us to acquire a large data set, minimising the statistical fluctuations, and at the same time distil as many signatures as possible from each acquired block. We estimate that this improves the standard QDS rate by about 10 times at short distances and even more at longer distances.

We start from analysing the MDI-QKD-mediated QDS rate on the link connecting Alice and Bob. Then, we will apply an analogous procedure in order to estimate the QDS rate on the QKD link between Alice and Charlie. The QDS protocol also includes a symmetrization step between Bob and Charlie performed on a secure channel (see Supplementary Note 1 for details)^19, 20^. This can be enabled by running QKD on the remaining link between Bob and Charlie. The specific key rate for this scheme would be the one showed in the bottom diagram of Fig. 3.

As a first QDS-specific quantity, we evaluate the minimum rate $p_{\rm{E}}^{{\rm{MDI}}}$ at which Eve can introduce errors on the MDI-QKD link. This is given by:

3 $$h\left( {p_{\rm{E}}^{{\rm{MDI}}}} \right) = \underline S _{{\rm{sig}}}^{{\rm{1,1}}}{\rm{/}}C_{{\rm{sig}}}^{s,s}\left[ {1 - h\left( {\overline e _{{\rm{ph,sig}}}^{1,1}} \right)} \right],$$

which is derived from Eq. (1), omitting the error correction and, for simplicity, the finite-size terms^20^. In Eq. (3), the subscript “sig” indicates that the quantities refer to the block from which signatures are extracted. In the QDS protocol, Alice randomly selects $C_{{\rm{sig}}}^{s,s} = 2.5 \times {10^6}$ bits from the *Z*-basis block, to form one of the signature blocks. Because this size is smaller than the Z-basis data set, she will be able to extract multiple signature blocks from it, all with size $C_{{\rm{sig}}}^{s,s}$. She then applies decoy-state estimation to find, for the signature block, the lower bound for Charlie’s counts due to single-photon pulses and the corresponding upper bound for the phase-error rate. In our experiment, the two bounds are $\underline S _{{\rm{sig}}}^{1,1} = 666,345$ bits and $\overline e _{{\rm{ph,sig}}}^{{\rm{1,1}}} = 0.053$, respectively, leading to $p_{\rm{E}}^{{\rm{MDI}}}$ = 0.0286.

The next step is to determine an upper bound for the QBER in the signature blocks, $\overline E _{{\rm{sig}}}^{s,s}$. For that, the QBER was directly measured on a sample set of $C_{{\rm{test}}}^{s,s} = 1,714,426$ bits and found to be $E_{{\rm{test}}}^{s,s} = 0.5\%$ (see also Supplementary Table 3). The measured block can be thought of as a random sample drawn from the overall *Z*-basis population. Therefore, the measured QBER is representative of the QBER in the non-measured fraction of the population. From this fraction, the users select several blocks of size $C_{{\rm{sig}}}^{s,s}$ to form the signatures. The QBER in each signature block is then estimated, by applying Serfling’s inequality^36^, to be:

4 $$\overline E _{{\rm{sig}}}^{s,s} = E_{{\rm{test}}}^{s,s} + \sqrt {\frac{{\left( {C_{{\rm{sig}}}^{s,s} + 1} \right)\left( {C_{{\rm{sig}}}^{s,s} + C_{{\rm{test}}}^{s,s}} \right)}}{{2 C_{{\rm{test}}}^{s,s}{{\left( {C_{{\rm{sig}}}^{s,s}} \right)}^{\!\!2}}}}{\rm{ln}}\left( {1{\rm{/}}{\epsilon _H}} \right)} .$$

The estimation in Eq. (4) provides $\overline E _{{\rm{sig}}}^{s,s} = 0.0085$ when we set ${\epsilon _H}$ = 2 × 10^−11^. After calculating suitable authentication and verification parameters, *s*_ab_ = 0.0152 and *s*_vb_ = 0.0219, we obtain the length of a signature $L_{{\rm{sig}}}^{{\rm{MDI}}}$ by inverting the relation

5 $$P_{{\rm{rep}}}^{{\rm{MDI}}} \le {\rm{exp}}\left[ { - {{\left( {{s_{{\rm{vb}}}} - {s_{{\rm{ab}}}}} \right)}^2}L_{{\rm{sig}}}^{{\rm{MDI}}}{\rm{/}}4} \right] \le 0.5 \times {10^{ - 10}},$$

which sets the repudiation probability $P_{{\rm{rep}}}^{{\rm{MDI}}}$ to less than 0.5 × 10^−10^ ^19, 20^. The resulting value for $L_{{\rm{sig}}}^{{\rm{MDI}}}$ is 2.11 × 10^6^, which is smaller than the set value of $C_{{\rm{sig}}}^{s,s}$, showing that Eq. (5) holds in our experiment when we take $C_{{\rm{sig}}}^{s,s}$ as the signature length. The overall failure probability at the end of the QDS distillation is less than 10^−10^, which is orders of magnitude smaller than in previous experiments^33–35^.

A signature of size $C_{{\rm{sig}}}^{s,s}$ can be generated with our system in 45 s on average. This is a remarkable speed for MDI-QKD-mediated QDS, if the increased security level entailed by the MDI-QKD link, by the lower failure probability and by the finite-size security against the most general attacks is taken into account. The average time results from the ratio of the total acquisition time in an experiment with an 80:20 bias between the *Z* and the *X* bases divided by the 1974 different signatures generated from the acquired data block. The reported average time includes the QKD operations on the other two links.

The analysis of MDI-QKD-based signatures is completed by calculating the probabilities of honest abort, *P*_hab_, and forging, *P*_for_, which are confirmed to be much smaller than the set threshold 10^−10^ with our experimental parameters.

As an additional step in the QDS scheme, we now evaluate the QDS rate on the 25-km QKD link between Alice and Charlie (see Fig. 4). We repeat similar calculations as for the MDI-QKD link. We set the size of the signature block to $C_{{\rm{sig}}}^s = 150,\!000$ bits, randomly selected in the *Z*-basis data block acquired by operating QKD on the AC link. In the signature block, the lower bound for Charlie’s counts due to single-photon pulses amounts to $\underline S _{{\rm{sig}}}^{\rm{1}} = 86,\!563$ and the upper bound for the phase-error rate is $\overline e _{{\rm{ph,sig}}}^{\rm{1}} = 0.0237$, leading to $p_{\rm{E}}^{^{{\rm{QKD}}}}$ = 0.105.

The actual QBER in the *Z* basis was measured on a sample of 46, 979, 354 bits and amounts to $E_{{\rm{test}}}^s = 0.0017$ (see also Supplementary Table 2). From it, using an equation similar to Eq. (4), we obtain an upper bound $\overline E _{{\rm{sig}}}^s = 0.0108$, which is less than $p_{\rm{E}}^{^{{\rm{QKD}}}}$, thus providing a positive QDS rate. To determine the rate, we calculate *s*_ac_ = 0.0421 and *s*_vc_ = 0.0734 and obtain a signature length $L_{{\rm{sig}}}^{{\rm{QKD}}} = 103,336$ by inverting an equation similar to Eq. (5), but with $p_{{\rm{rep}}}^{{\rm{QKD}}}$ replacing $p_{{\rm{rep}}}^{{\rm{MDI}}}$. The total repudiation probability is then given by the sum $p_{{\rm{rep}}}^{{\rm{QKD}}} + p_{{\rm{rep}}}^{{\rm{MDI}}}$.

The total time the system would spend acquiring QKD data on the AC link is about 36 s. The resulting data block would be enough to distil signatures for 2506 1-bit messages, thus providing an average time for the QKD-only operations on the AC link equal to 72 ms for each signed bit. A similar value of 74 ms could have been obtained on the QKD link between Bob and Charlie if we had used it to distil signatures rather than for encryption. Although the reported values can be further improved by optimising the initial parameters set by Alice and Bob, they are already in line with state-of-the-art QDS, especially when considering the fact that the present scheme offers security in the finite-size scenario against the most general attack allowed by the laws of physics.

### Real-world implementation

As a final point, let us briefly discuss the possibility to export our methods to a real-world platform. Our quantum network features a majority of QKD links, used to distil information-theoretically secure encryption keys and digital signatures for the network’s users. This would ease the transition to a real-world system, as QKD offers excellent stability^37^ and has already been implemented in field trials^38–40^. The polarisation encoding used in our setup has been used in previous quantum communications experiments and is controllable with high accuracy^41^. The polarisation drift is negligible in our laboratory for the duration of the experiment. Similar or more favourable conditions are expected in underground fibres^42^, where the polarisation drift is not a limiting factor. The necessary synchronisation of the signals has been demonstrated in the QKD field trials as well as in an MDI-QKD network^13^. The effect of stray photons due to the multiplexing of classical signals can be also effectively handled, either using two different fibres^37^ or multiplexing the clock and data signals with the quantum signals. Distances up to 200 km have been reached in this configuration^43^. Finally, the stringent mode-matching conditions necessary for high-speed MDI-QKD have been recently considerably relaxed using laser seeding^44, 45^, which is the same enabling technology adopted in this work (see Fig. 2). For these reasons, we would not expect a major reduction of the rates presented in Fig. 3 if we turned our setup into a prototype suitable for long-term deployment in the field, as it contains already the necessary enabling technology.

## Methods

### Experimental details

Alice and Bob use the experimental setup shown in Fig. 2 to independently produce 32 ps pulses at 1549.8 nm. The two parties create low-jitter (1.7 ps) signals using pulsed laser seeding. To enable this, the master and slave laser in each source are driven by an AC bias and a DC bias. The AC biases are equal, however the DC bias for the master laser is higher than that of the slave laser. This ensures that the master laser has a shorter turn-on time, emitting longer pulses of around 250 ps. The master laser is input to the slave via a circulator and the AC bias is temporally offset between the two lasers to ensure injection occurs at the correct time. Each of the laser diodes are independently temperature controlled to ensure the emission wavelengths are stable and identical. The 1 GHz gain switching of both lasers ensures that all pulses are perfectly phase randomised.

Alice and Bob’s pulses are then passed through 30 GHz bandwidth filters to remove noise. The polarisation of the pulses is controlled using an electric polarisation controller, which can create all of the required polarisation states. The polarisation is set once prior to each measurement using Charlie’s detectors as a reference for each polarisation state. The polarisation drift is negligible for the duration of the data acquisition due to the controlled temperature of the laboratory.

An attenuator provides the four photon fluxes (*s*, *u*, *v*, *w*) before they are sent to Charlie. Following this, fixed optical attenuators simulate a lossy channel at all distances reported in Fig. 3, assuming the loss rate is 0.2 dB/km as in a standard single-mode optical fibre in the third telecom window. In one case, we replaced attenuators with real optical fibre. The star points in Fig. 3 were obtained using two 25-km reels of standard optical fibre, one connecting Alice’s setup to Charlie and the other connecting Bob to Charlie. The photon flux of the *u* state is set for each distance to produce 5.8 × 10^6^ photons/s at Charlie’s detectors to avoid saturation of the single-photon counter. An emulator incorporating finite-key size analysis is then used to determine the optimal photon fluxes of the other states to maximise the MDI-QKD key rate.

Alice, Bob and Charlie share a common 10-MHz reference clock. This is then regenerated to the master clock frequency of 1 GHz at each of the users. This allows Alice and Bob to precisely overlap their optical pulses on Charlie’s BS and at the same time permits Charlie to time align the detector gates to the received optical pulses. This is the same method employed in QKD experiments^37, 43^ and QKD field trials^39, 43^ to distribute the clock between the users. In some cases^37^, the clock is distributed using two different fibres. In other cases^43^, the clock is multiplexed with the quantum channel in the same optical fibre.

At Charlie’s side, the photons from Alice and Bob interfere on a BS. One output is incident on a PBS and the other output passes through a fixed 45° polarisation rotator before travelling to a different PBS. With this design, one arm detects photons in the rectilinear basis and the other arm detects photons in the diagonal basis. The overall insertion loss of Charlie’s setup up to this point is 1 dB. The outputs from the PBS’s are detected by four InGaAs self-differencing avalanche photodiodes. These detectors are gated at 1 GHz and have an intrinsic deadtime of 1 ns. They feature an average efficiency of 20.9%, a dark count rate of 16 kHz and an afterpulsing probability of 3.9%. They are maintained at a temperature of 273 K for all measurements. The resultant signals, when neither party is transmitting in the *Z* basis, are counted in real time using a multiple-event time digitizer with 100 ps time bins and a saturation value of 6.5 × 10^6^ counts/s. Counts when either party, or both parties, are sending the *Z*-basis state are detected on an oscilloscope and analysed with Matlab because the high-photon levels would saturate the digitizer. The single counts, coincidence counts and transmitted photon fluxes are collected at each distance for analysis.

### Data availability

The data that support the findings of this study are available from the corresponding author on reasonable request.

## Electronic supplementary material

Supplementary Information
