# Supplementary material for: Experimental measurement-device-independent quantum digital signatures
Source: Nat Commun. 2017 Oct 23;8:1098. doi: 10.1038/s41467-017-01245-5 (PMC5653667; doi:10.1038/s41467-017-01245-5)
Supplement: Supplementary file 1 — Supplementary Information [file 41467_2017_1245_MOESM1_ESM.pdf]

Attenuation A-B [Distance A-B]	Alice - Bob MDI-QKD (kbps)	Alice - Charlie QKD (kbps)	Bob - Charlie QKD (kbps)
0 dB [0 km]	134.006	4,854.663	4,821.267
6 dB [30 km]	21.701	2,553.560	2,722.042
10 dB [50 km, real fibre]	7.738	1,200.618	1,322.767
12 dB [60 km]	5.332	1,151.439	1,326.426
18 dB [90 km]	0.606	489.127	464.659

Supplementary Table 1: **Key Rates**. Secure key rates for MDI-QKD and QKD. The equivalent distance assumes 0.2 dB/km attenuation in a single mode optical fibre and refers to the link between Alice and Bob. The distance of the QKD links connected to Charlie are half Alice-Bob distance.

Attenuation [Distance]		Alice - Charlie (Bob prepares w_2)		Bob - Charlie (Alice prepares w_2)		Intensity (ph/pulse)	Acq. Time (s)
		Single counts	QBER (%)	Single counts	QBER (%)		
0 dB [0 km]	s	206,390,665	0.08	194,087,965	0.07	0.7400	8
	u	307,255,370	1.79	276,655,995	2.67	0.0159	1200
	v	175,887,345	3.31	163,487,050	3.78	0.0091	1200
	w_1	9,842,760	30.37	9,772,650	40.87	0.0001	1200
3 dB [15 km]	s	100,024,781	0.11	100,798,034	0.09	0.6900	8
	u	292,849,155	1.57	297,798,630	1.73	0.0327	1200
	v	163,546,220	2.60	170,059,925	2.71	0.0180	1200
	w_1	7,788,060	50.00	7,718,620	49.99	0.0001	1200
5 dB [25 km Real fibre]	s	46,979,354	0.17	45,707,894	0.24	0.6200	8
	u	224,105,855	1.52	216,126,660	1.61	0.0597	1200
	v	116,311,785	2.77	118,991,520	2.55	0.0277	1200
	w_1	5,367,381	41.89	5,531,287	45.76	0.0001	1200
6 dB [30 km]	s	45,227,196	0.19	46,490,029	0.16	0.6000	8
	u	293,856,075	1.48	308,237,415	1.52	0.0661	1200
	v	144,852,015	2.67	155,242,665	2.49	0.0322	1200
	w_1	6,743,450	43.21	6,775,920	49.74	0.0001	1200
9 dB [45 km]	s	15,393,126	0.48	16,214,775	0.45	0.4000	8
	u	296,471,815	1.36	284,181,140	1.64	0.1262	1200
	v	132,471,231	2.67	126,388,566	2.86	0.0551	1200
	w_1	6,050,370	45.28	6,123,407	49.77	0.0001	1200

Supplementary Table 2: **QKD counts and error rates**. Measured single counts in matched bases and QBER on Alice-Charlie and Bob-Charlie links. For the intensities u, v and w, all encoded in the same basis as w, the single counts have been measured together with the MDI-QKD coincidence counts (see Table 3), to demonstrate the reconfigurability property of the scheme. For the intensity s, which belongs to a different basis than w (Z instead of X), the counts have been measured in a separate experiment. The time reported in the last column is the sum of the acquisition times for Alice-Charlie and Bob-Charlie. The number of coincidence counts is less than 0.1% of the single counts.

Attenuation [Distance]		Coincidence counts in H/V detectors				QBER (%)				Intensity (ph/pulse)	Acq. Time (s)
		s	u	v	w	s	u	v	w		
0 dB [0 km]	s	509,354				0.06				0.7400	0.4
	u		759,198	484,304	209,303		27.92	29.46	48.64	0.0159	1,200
	v		463,303	251,526	69,084		28.66	27.80	48.05	0.0091	1,200
	w		185,962	65,235	307		48.47	47.97	49.51	0.0001	1,200
6 dB [30 km]	s	138,208				0.22				0.6900	0.4
	u		788,410	467,698	189,563		28.15	29.64	49.85	0.0327	1,200
	v		479,408	247,588	61,076		30.41	28.35	49.08	0.0180	1,200
	w		215,869	70,665	181		49.91	49.81	49.17	0.0001	1,200
10 dB [50 km Real fibre]	s	1,714,426				0.50				0.6200	20
	u		759,224	434,458	183,525		28.19	30.90	49.90	0.0597	1,200
	v		396,914	189,286	52,019		32.19	28.70	43.72	0.0277	1,200
	w		204,026	52,967	121		49.56	52.27	41.32	0.0001	1,200
12 dB [60 km]	s	28,209				0.21				0.6000	0.4
	u		783,972	432,905	189,135		28.15	30.21	49.52	0.0661	1,200
	v		452,767	194,339	46,014		31.25	28.32	49.57	0.0322	1,200
	w		223,386	57,035	142		49.63	49.34	49.30	0.0001	1,200
18 dB [90 km]	s	1,160				0.68				0.4000	0.4
	u		668,322	351,779	177,526		28.23	34.87	49.87	0.1262	1,200
	v		339,422	132,089	35,767		31.52	30.51	49.92	0.0551	1,200
	w		172,026	35,466	93		46.65	48.23	42.50	0.0001	1,200

Supplementary Table 3: **MDI-QKD count and error rates**. Measured coincidence counts and QBER in the MDI-QKD modality.

Supplementary Note 1 - Quantum protocols

In this section we list all the protocols related to the main manuscript, including the one used for switching between QKD and MDI-QKD in the reconfigurable scheme, those used to generate keys from QKD and MDI-QKD with the 4-intensity protocol and the one for distilling multiple QDS from the same data block in the finite-size scenario.

Reconfigurable MDI/QKD

In order to switch between QKD and MDI-QKD in the reconfigurable scheme, the users perform the following steps using the setup in Fig. 1:

Reconfigurable MDI/QKD network

---

1. Alice and Bob randomly choose the bit value, 0 or 1, with probability 50% each, and the basis, Z or X, with probabilities $p_Z = 80\%$ and $p_X = 20\%$, respectively (In the protocol, we fix all the probability values for simplicity. However we do not claim that the set values are optimal. Other settings are possible and can improve the overall performance). They also choose the intensity of the pulses, s (signal), u (decoy 1), v (decoy 2) and w (vacuum). If the basis Z is selected, then they prepare the intensity s with probability $p_{s|Z} = 1$. If the basis X is selected, they prepare one of the intensities u, v or w, with probabilities close to unbiased: $p_{u|X} = p_{v|X}$=33%, $p_{w|X} = 34\%$. The modality "Stop" in Fig. 1 corresponds to setting the intensity modulator to prepare the intensity w.

2. We write the probability of the vacuum state as $p_w = p_{w_1} + p_{w_2}$, with $p_{w_1} = 33\%$ and $p_{w_2} = 1\%$, and set $w_1 = w_2 = w$. The values of p_{w_1} and p_{w_2} are set to make the key rates of MDI-QKD and QKD more balanced. Different values can be chosen to optimise the performance. If, in a given run, Alice (Bob) selected w_2 and Bob (Alice) did not select w_2, that run is assigned to the QKD between Charlie and Bob (Alice). In all the other cases it is assigned to MDI-QKD. Supplementary Table 4 helps understand the mechanism used for the assignments.

3. Alice and Bob send out the prepared optical pulses. Charlie measures all the pulses and records the result.

4. After the quantum transmission is over, Alice and Bob reveal the runs where they prepared w_2. Based on this announcement, all parties assign each run to QKD or MDI-QKD, according to the above Step 2.

Alice \ Bob	(s, p_s)	(u, p_u)	(v, p_v)	(w_1, p_{w_1})	(w_2, p_{w_2})
(s, p_s)	MDI	MDI	MDI	MDI	QKD-AC
(u, p_u)	MDI	MDI	MDI	MDI	QKD-AC
(v, p_v)	MDI	MDI	MDI	MDI	QKD-AC
(w_1, p_{w_1})	MDI	MDI	MDI	MDI	QKD-AC
(w_2, p_{w_2})	QKD-BC	QKD-BC	QKD-BC	QKD-BC	MDI

Supplementary Table 4: **Communication protocol distribution**. Distribution of the preparations between MDI-QKD for distilling a key between Alice and Bob, QKD for distilling a key between Alice and Charlie and QKD for distilling a key between Bob and Charlie. At all distances we set $p_s = p_Z \times p_{s|Z} = 80\% \times 100\%$, $p_u = p_X \times p_{u|X} = 20\% \times 33\% = 6.6\% = p_v = p_{w_1}$, and $p_{w_2} = p_X \times p_{w_2|X} = 20\% \times 1\% = 0.2\%$.

QKD in reconfigurable MDI/QKD

In all the runs assigned to QKD, Charlie and one of the remaining users will be running the BB84 protocol [1]. For definiteness, we focus on the user Alice in what follows, communicating with Charlie on the AC link. However, all the conclusions hold by replacing Alice with Bob and the AC channel with the BC channel. The BB84 protocol is implemented with a passive choice of the basis on Charlie's side, performed by his 50/50 BS (see Fig. 1), and an active choice of the basis on Alice's side, performed using her PM (see Figs. 1 and 2). The key distillation protocol runs as follows:

QKD protocol (encryption)

---

1. Charlie announces the runs in which at least one of his detectors fired. All the runs where no detector fired are discarded. Charlie applies the following subroutine to enable the "squashing model" [2, 3, 4] for the BB84 protocol, which accounts for the runs where multiple detectors fired:

   (a) If a single detector clicks, that detector determines Charlie's basis and bit values, which we denote by σ and τ, respectively. Charlie records the pair (σ, τ) and proceeds to the next clock.

   (b) If two detectors belonging to the same basis click, i.e., H/V or D/A in Fig. 1 of the main text, those detectors determine Charlie's basis, σ. In this case, the bit value τ is randomly assigned by Charlie to either 0 or 1. Then he records the pair (σ, τ) and moves on.

   (c) If two detectors belonging to different bases click, i.e., H/D, H/A, V/D or V/A in Fig. 1 of the main text, Charlie chooses the basis value σ at random between Z and X. Then, for the chosen basis, the bit value τ is determined by the label on the detector that clicked. Charlie records the pair (σ, τ) and moves on.

(d) If three detectors click, then one of them belongs to a certain basis, e.g. Z, and the other two belong to the other basis, e.g. X. In this case, Charlie assigns the basis value σ at random between Z or X. If the selected basis contains only one detector that clicked, then the bit value τ is assigned by the label on that detector. If the selected basis contains two detectors that clicked, then the bit value τ is chosen at random by Charlie. He then records the pair (σ, τ) and proceeds to the next run.

(e) If four detectors click, then Charlie assigns randomly both the basis and the bit values, records the pair (σ, τ) and move on to the next clock.

2. Alice and Charlie announce the bases and discard all the runs where they are not matching. For the matching basis X (test basis), they also disclose all the bit values, whilst keeping secret the bit values in the Z basis (data basis).

3. Alice announces the intensity settings in the X basis.

4. The users run the decoy-estimation routine in the X basis and calculate lower bounds for the single photon yield y_X^1 and single-photon QBER e_X^1. From these values, the users estimate the amount of privacy amplification (PA) needed to remove any residual Eve's information on the bits in the Z basis.

5. The users run error correction (EC) and PA on the bits of the Z basis to obtain the final secure key and the error verification procedure to guarantee the correctness of the key.

The final key rate associated with the above protocol was deduced using the method in [5] and in the main text it was given as

$$R^{\mathrm{QKD}} = \underline{S}^1[1 - h(\overline{e}_{\mathrm{ph}}^1)] - leak_{\mathrm{EC}}^{\mathrm{QKD}} - \Delta^{\mathrm{QKD}}. \tag{1}$$

The quantity $leak_{\mathrm{EC}}^{\mathrm{QKD}}$ is due to the amount of EC performed in the system. This is a directly measurable quantity in any QKD experiment. In our case, we measured the counts C_Z^s and the QBER E_Z^s associated with the signal states sent by Alice conditional on Bob preparing w_2 (see Table 3), and calculated the product $C_Z^s \times f_{\mathrm{EC}} \times h(E_Z^s)$, where $f_{\mathrm{EC}} = 1.16$ is the inefficiency of EC and h is the binary entropy function. The quantity Δ^{QKD} takes into account the finite-size effect and amounts to $6 \log_2 \frac{21}{\epsilon_{\mathrm{sec}}} + \log_2 \frac{2}{\epsilon_{\mathrm{cor}}}$, where ϵ_{sec} is the overall failure probability of the system, set equal to 10^{-10}, and ϵ_{cor} is the correctness parameter, set equal to 10^{-15}. This expression has been derived from Eq. (B4) of [5], after modifying it to take into account the actual number of constraints used in our parameter estimation procedure, as explained below.

The parameter estimation procedure aims at calculating the single-photon quantities S^1 and e_{ph}^1 and it needs to be extended from the protocol presented in [5] because we make use of four intensities rather than three in our implementation. However, we still use three intensities in the

X basis. Therefore we can at once calculate the single-photon quantities in the X basis using the equations in [5] specific to the X basis:

$$\underline{S}_X^1 = \frac{u\ \tau_{X,1}}{u(v-w)-v^2+w^2}\left(\frac{e^v\underline{C}_X^v}{p_{v|X}} - \frac{e^w\overline{C}_X^{w_1}}{p_{w_1|X}} - \frac{v^2-w^2}{u^2}\frac{e^u\overline{C}_X^u}{p_{u|X}}\right) \tag{2}$$

In Eq. (2), $\underline{S}_X^1$ is the lower bound for the number of detections by Charlie in the X basis due to single-photon pulses. The probabilities to encode u, v and w_1 conditional on Alice choosing the X basis and Bob preparing the w_2 state are $p_{u|X} = p_{v|X} = p_{w_1|X} = 33\%$ (see also Table 1 in Sec.). The term $\tau_{X,1} = ue^{-u}p_{u|X} + ve^{-v}p_{v|X} + we^{-w}p_{w_1|X}$ is the probability that Alice sends out a single-photon pulse conditional on her choosing one of the X basis states u, v or w_1 and Bob preparing the w_2 state. An analogous quantity $\tau_{Z,1}$ can be defined in the Z basis for the signal state s. Finally, C_X^u, C_X^v and $C_X^{w_1}$ are Charlie's counts for the preparations u, v and w_1, respectively. The upper and lower bars indicate upper and lower bounds for these quantities, which are obtained by applying Hoeffding's double-tail inequality to the set of detected counts [6, 5]. According to that, each bound can fail with probability 2ϵ, with ϵ a positive real number chosen equal to $10^{-10}/21$. Because three such bounds are present in Eq. (2), the overall failure probability in the estimation of $\underline{S}_X^1$ amounts to 6ϵ.

The next step is estimating the upper bound for the single-photon QBER in the X basis. This is given by $\overline{e}_X^1 = \overline{T}_X^1/\underline{S}_X^1$, where $\overline{T}_X^1$ is the upper bound for the bit errors found in the X basis in the set of single-photon detections, given by [5]:

$$\overline{T}_X^1 = \frac{\tau_{X,1}}{v-w}\left(\frac{e^v\overline{D}_X^v}{p_{v|X}} - \frac{e^w\underline{D}_X^{w_1}}{p_{w_1|X}}\right). \tag{3}$$

The quantities in Eq. (3) are analogous to Eq. (2), with D_X^v and $D_X^{w_1}$ denoting the errors found in Charlie's counts for the preparations v and w_1, respectively. The upper and lower bars indicate upper and lower bounds, obtained by applying Hoeffding's inequality [5, 6], to the designated quantities. The overall contribution to the security parameter from the estimation of $\overline{T}_X^1$ is 4ϵ, due to the presence of two bounds in Eq. (3).

The final step is moving from the above single-photon parameters estimated in the X basis to those in the Z basis, which form the final key. In the following, we sketch how to do this for the QKD protocol. A similar approach was already presented for MDI-QKD in [7].

To move from one basis to the complementary one, we extend to the finite-size scenario the following relations, which hold for the BB84 protocol in the asymptotic scenario in absence of implementation imperfections:

$$y_Z^1 = y_X^1, \tag{4}$$

$$e_{\mathrm{ph},Z}^1 = e_X^1. \tag{5}$$

The first equation states that the single-photon yield is basis independent, whereas the second is an expression of the uncertainty principle, stating that in the BB84 protocol the single photon phase error rate in the Z basis is equal to the single photon bit error rate in the X basis. By virtue of these equations, we can interpret the Z-basis counts as the result of a random sampling operation from the total population of single-photon pulses emitted by Alice.

By applying Hoeffding's inequality [6], we can first bound the total population of single-photon preparations. The lower bound for the Z basis population is given with confidence $1 - \epsilon_{1z}$ by $\underline{n}_Z^1 = se^{-s}N_Z - \sqrt{N_Z/2\ln(1/\epsilon_{1z})}$, with N_Z counting the total runs where Alice and Charlie chose the basis Z. The upper bound for the X basis population is given with confidence $1 - \epsilon_{1x}$ by $\overline{n}_X^1 = \sum_{i=\{u,v,w\}} \overline{n}_{X,i}^1$, with $\overline{n}_{X,i}^1 = ie^{-i}N_{X,i} + \sqrt{N_{X,i}/2\ln(1/\epsilon_{1x,i})}$, $N_{X,i}$ counting the total runs where Alice and Charlie chose the basis X and Alice prepares the intensity $i = \{u, v, w\}$, $\epsilon_{1x,i}$ the error probability associated with the estimation of $\overline{n}_{X,i}^1$ and $\epsilon_{1x} = \sum_{i=\{u,v,w\}} \epsilon_{1x,i}$.

Because of the basis independence of the counts, we can now interpret the basis choice in the whole population of the single-photon preparations as an operation of sampling without replacement. Therefore we can use Serfling's inequality [8] to lower bound with confidence $1 - \epsilon_z$ the single-photon counts in the Z basis [9]:

$$\underline{S}_Z^1 = \underline{S}_X^1 \times \frac{\underline{n}_Z^1}{\overline{n}_X^1} - \sqrt{\frac{(\underline{n}_Z^1 + 1)(\overline{n}_X^1 + \underline{n}_Z^1)}{2\overline{n}_X^1}\ln(1/\epsilon_z)}. \tag{6}$$

Now we can repeat similar steps for the phase-error rate in the Z basis. We identify the overall population as $\underline{S}_Z^1 + \underline{S}_X^1$ and we perform a random sampling without replacement in this population to estimate the amount of phase errors $\overline{T}_{\text{ph},Z}^1$. Un upper bound for this quantity can be obtained with confidence $1 - \tilde{\epsilon}_z$ by means of Serfling's inequality [8]:

$$\overline{T}_{\text{ph},Z}^1 = \overline{T}_X^1 \times \frac{\underline{S}_Z^1}{\underline{S}_X^1} + \sqrt{\frac{(\underline{S}_Z^1 + 1)(\underline{S}_X^1 + \underline{S}_Z^1)}{2\underline{S}_X^1}\ln(1/\tilde{\epsilon}_z)}. \tag{7}$$

The upper bound for the phase error rate in the Z basis is eventually given by:

$$\overline{e}_{\text{ph},Z}^1 = \frac{\overline{T}_{\text{ph},Z}^1}{\underline{S}_Z^1}, \tag{8}$$

and the overall error probability of the protocol amounts to $\epsilon_{\text{tot}} = 2 \times (\epsilon_{1z} + \epsilon_z + \epsilon_{1x} + 6\epsilon) + \tilde{\epsilon}_z + 4\epsilon = 1.185 \times 10^{-10}$, with $\epsilon_{1z} = \epsilon_z = \tilde{\epsilon}_z = 5 \times 10^{-12}$, $\epsilon_{1x} = 1.5 \times 10^{-11}$ and $\epsilon = 10^{-10}/21$.

We finally notice that the Z subscript in the above equations has been dropped in the main text for readability purposes.

QKD\EP in reconfigurable MDI/QKD

This protocol runs exactly the same as the QKD protocol described in the previous section, up to a certain point, which we specify here. Essentially, the participants of the protocol only perform the quantum part of the QKD protocol to generate different raw keys, and do not perform error correction and privacy amplification. These keys are bit strings which are imperfectly correlated and not completely secret. This is adequate for quantum signatures [10, 11]. For definiteness, in the description we focus again on the users Alice and Charlie on the AC link. However, all the conclusions hold for the users Bob and Charlie on the BC channel as well.

QKD\EP protocol (signature)

---

1. - 5. Same steps as in QKD protocol. From these steps, the users get the bounds for the single-photon quantities using the methods described in Sec. , in particular $\underline{S}_Z^1$ and $\overline{e}_{\mathrm{ph},Z}^1$.

6. From the total counts in the Z basis the users select a random subset of C_{test}^s bits and measure the QBER E_{test}^s in this subset.

7. The remaining counts in the Z basis are randomly grouped into m_{sig} subsets, each of size C_{sig}^s. From the concatenation of the bits in each subset, the users will form m_{sig} digital signatures.

8. The users estimate an upper bound for the QBER in the signatures subsets, $\overline{E}_{\mathrm{sig}}^s$ using the measured value of E_{test}^s and Serfling's inequality, through an equation similar to Eq. (3) in the main text, written below as Eq. (9).

9. The users estimate the bounds for the single-photon quantities in the signatures subsets, in particular $\underline{S}_{\mathrm{sig}}^1$ and $\overline{e}_{\mathrm{ph,sig}}^1$, using Eqs. (10) and (11), respectively, reported below.

10. The users estimate all the parameters relevant to the quantum digital signatures from the equations given below and the procedure described in the main text.

Eq. (9) mentioned in Step 8 of the above protocol is about the maximum of the worst-case QBER Alice estimates on Charlie's key, $\overline{E}_{\mathrm{sig}}^s$, given by

$$\overline{E}_{\mathrm{sig}}^s = E_{\mathrm{test}}^s + \frac{1}{C_{\mathrm{sig}}^s}\sqrt{\frac{(C_{\mathrm{sig}}^s + 1)(C_{\mathrm{sig}}^s + C_{\mathrm{test}}^s)}{2\ C_{\mathrm{test}}^s}\ln(1/\epsilon_H)}. \tag{9}$$

It stems from Serfling's inequality [8] applied to the data sample formed by $C_{\mathrm{test}}^s + C_{\mathrm{sig}}^s$, in which E_{test}^s is known because it is directly measured. The inequality in Eq. (9) entails an overall failure probability ϵ_H, the value of which is set equal to $\epsilon_{\mathrm{sec}} = 2 \times 10^{-11}$.

Eq. (10) mentioned in Step 9 of the above protocol aims at estimating the amount of single-photon detections in a signature subset. From Step 4 of the protocol, the users estimate the minimum number of single-photon detections $\underline{S}_Z^1$ in the Z basis data sample, the size of which has already been defined as C_Z^s. Therefore, because the $C_{\rm sig}^s$ bits of the signature subset are randomly selected from the Z basis sample, we can apply a simple proportionality rule and Serfling's inequality [8] to obtain

$$\underline{S}_{\rm sig}^1 = \underline{S}_Z^1 \times \frac{C_{\rm sig}^s}{C_Z^s} - \sqrt{\frac{(C_Z^s - C_{\rm sig}^s)(C_{\rm sig}^s + 1)}{2C_Z^s} \ln(1/\epsilon_H)}. \tag{10}$$

By the same argument, we can apply Serfling's inequality again to estimate the upper bound for the phase-error rate $\overline{e}_{\rm ph,sig}^1$ in the population $\underline{S}_{\rm sig}^1$ starting from the phase error rate $\overline{e}_{{\rm ph},Z}^1$ in the overall population $\underline{S}_Z^1$,

$$\overline{e}_{\rm ph,sig}^1 = \overline{e}_{{\rm ph},Z}^1 + \frac{1}{\underline{S}_{\rm sig}^1} \sqrt{\frac{(\underline{S}_Z^1 - \underline{S}_{\rm sig}^1)(\underline{S}_{\rm sig}^1 + 1)}{2\ \underline{S}_Z^1} \ln(1/\epsilon_H)}. \tag{11}$$

Eqs. (10) and (11) contribute to the overall failure probability of the signature distillation protocol by a factor ϵ_H each. By adding up these two failure probabilities, the one related to Eq. (9) and the one related to the decoy-state parameter estimation, $\epsilon_{\rm sec}$, we obtain a total failure probability less than 10^{-10}.

From the quantities specified above, the users can estimate the probability $p_E^{\rm QKD}$ mentioned in the main text by solving the following equation:

$$h\left(p_E^{\rm QKD}\right) = \frac{\underline{S}_{\rm sig}^1}{C_{\rm sig}^s}[1 - h(\overline{e}_{\rm ph,sig}^1)], \tag{12}$$

where $p_E^{\rm QKD}$ represents the minimum adversary's error rate on the Alice-Charlie QKD\EP link.

The correctness and security of the protocol depend on the choice of the parameters s_{ac} and s_{vc} (see main text and Sec.). They are chosen such that $\overline{E}_{\rm sig}^s < s_{ac} < s_{vc} < p_E^{\rm QKD}$.

Using these parameters we calculate the length of the signature, $L_{\rm sig}^{\rm QKD}$, necessary to sign a message with a security level of 10^{-10}. This is achieved by inverting the following relation, which determines the repudiation probability,

$$P_{\rm rep}^{\rm QKD} \leq \exp\left[-(s_{vc} - s_{ac})^2 L_{\rm sig}^{\rm QKD}/4\right] \leq 0.5 \times 10^{-10}. \tag{13}$$

From [10, 11], we obtain the probabilities of honest abort ($P_{\rm hab}^{\rm QKD}$) and forging ($P_{\rm for}^{\rm QKD}$), given by

$$P_{\rm hab}^{\rm QKD} \leq 2\epsilon_H, \tag{14}$$

$$P_{\text{for}}^{\text{QKD}} \leq a + \epsilon_F + \epsilon_H + \epsilon_{\text{tot}}. \tag{15}$$

Here, ϵ_F is given by

$$\epsilon_F := \frac{1}{a}\left(2^{-\frac{L_{\text{sig}}^{\text{QKD}}}{2}\left\{\frac{2\underline{S}_{\text{sig}}^1}{L_{\text{sig}}^{\text{QKD}}}[1-h(\overline{e}_{\text{ph,sig}}^1)]-h(s_v)\right\}} + \epsilon\right). \tag{16}$$

The value of a is set equal to ϵ_H and Eq. (15) is valid for any choice of a, $\epsilon_H > 0$ and thereby can be made arbitrarily small by increasing $L_{\text{sig}}^{\text{QKD}}$.

MDI-QKD in reconfigurable MDI/QKD

Whenever the MDI-QKD modality is enabled in the execution of the MDI/QKD reconfigurable network, the users run the efficient MDI-QKD protocol described in Sec. A of Supplementary Information of [7], with the difference that only the coincidence counts coming from detectors H and V in Fig. 1 of the main text are used, whereas all the other counts are discarded. For self-completeness we list the steps of the protocol below, referring the reader to [7] for the details.

MDI-QKD protocol (encryption)

1. Charlie announces the runs where only his two detectors H and V fired in coincidence.

2. The users Alice and Bob assign these successful events to the triplet state, $|\psi^+\rangle = (|HV\rangle + |VH\rangle)/\sqrt{2}$ [12].

3. Alice and Bob announce their bases and intensities for the successful events. For the matching basis X (diagonal, or test, basis), they also disclose all their bit values, whilst keeping secret the bit values in the Z basis (rectilinear, or data, basis). Bob performs a bit flip of his bits in the rectilinear basis to match them to Alice's ones [12].

4. The users run the decoy-estimation routine in the test basis and calculate the lower bound for the single photon yield, $\underline{y}_X^{1,1}$, and the upper bound for the single-photon error rate, $\overline{e}_X^{1,1}$. This estimation makes use of the bounds $\underline{n}_X^{1,1}$ and $\overline{n}_X^{1,1}$ for the population of single-photon signals prepared by Alice and Bob in the test basis, obtained from Hoeffding's inequality with a procedure outlined below.

5. From the bounds obtained in the test basis, the users estimate in the data basis a lower bound for the single-photon yield, $\underline{y}_Z^{1,1}$, and an upper bound for the single-photon phase error rate, $\overline{e}_{\text{ph},Z}^{1,1}$. This new estimation exploits the bounds $\underline{n}_Z^{1,1}$ and $\overline{n}_Z^{1,1}$ for the population of single-photon signals prepared by Alice and Bob in the data basis, obtained from Hoeffding's inequality with a procedure outlined below.

6. The users run error correction (EC) and PA on the bits of the data basis to obtain the final secure key, and the error verification procedure to guarantee the correctness of the key.

The key rate for the above-described protocol is given in Eq. (1) of the main text, which we repeat here for convenience:

$$R^{\mathrm{MDI}} = \underline{S}^{1,1}[1 - h(\overline{e}^{1,1}_{\mathrm{ph}})] - leak^{\mathrm{MDI}}_{\mathrm{EC}} - \varDelta^{\mathrm{MDI}}. \tag{17}$$

The quantity $\underline{S}^{1,1}$ can be calculated from Step 5 of the protocol as $\lfloor\ \underline{y}^{1,1}_Z\ \underline{n}^{1,1}_Z\rfloor$. The quantity $leak^{\mathrm{MDI}}_{\mathrm{EC}}$ is due to the amount of EC performed in the system, directly measurable in any MDI-QKD experiment. In our case, we measured the counts $C^{s,s}_Z$ and the QBER $E^{s,s}_Z$ associated with the signal states sent by Alice and Bob (see Table 4), and calculated the product $C^{s,s}_Z \times f_{\mathrm{EC}} \times h(E^{s,s}_Z)$, where $f_{\mathrm{EC}} = 1.16$ characterises the inefficiency of EC and h is the binary entropy function. The quantity $\varDelta^{\mathrm{MDI}}$ takes into account the finite-size effect. It amounts to $\log_2(8/\epsilon_{\mathrm{cor}}) + 2\log_2[2/(\epsilon'\tilde{\epsilon})] - 2\log_2(2\epsilon_{\mathrm{PA}})$[9] and is very small when $\epsilon_{\mathrm{cor}} = 10^{-15}$, $\epsilon' = \tilde{\epsilon} = \epsilon_{\mathrm{PA}} = 3.875 \times 10^{-13}$, as in [7] and in the current implementation.

The parameter estimation procedure in the decoy-state MDI-QKD protocol is executed in Step 4 of the protocol. It uses a numerical routine optimised for the case where Alice and Bob prepare four intensities [7, 13], as in the present case, and returns the bound $\underline{y}^{1,1}_X$ with confidence $1 - \epsilon_{y_x}$ and the bound $\overline{e}^{1,1}_X$ with confidence $1 - \epsilon_{e_x}$. In the estimation routine, the worst-case bounds $\overline{n}^{1,1}_X$ and $\underline{n}^{1,1}_X$ are obtained using Hoeffdding's inequality [6]. Explicitly they are given with confidence $1 - \epsilon_{11x}$ by

$$\overline{n}^{1,1}_X = \sum_{\{i,j\}=\{u,v,w\}} \overline{n}^{1,1}_{X,i,j}, \tag{18}$$

$$\underline{n}^{1,1}_X = \sum_{\{i,j\}=\{u,v,w\}} \underline{n}^{1,1}_{X,i,j}, \tag{19}$$

with

$$\overline{n}^{1,1}_{X,i,j} = ije^{-(i+j)}N_{X,i,j} + \sqrt{N_{X,i,j}/2\ln(1/\epsilon_{11x,i,j})}, \tag{20}$$

$$\underline{n}^{1,1}_{X,i,j} = \max\left[ije^{-(i+j)}N_{X,i,j} - \sqrt{N_{X,i,j}/2\ln(1/\epsilon_{11x,i,j})}, 1\right]. \tag{21}$$

The quantity $N_{X,i,j}$ counts the total runs where Alice and Bob choose the basis X, Alice (Bob) prepares the intensity $i = \{u, v, w\}$ ($j = \{u, v, w\}$), $\epsilon_{11x,i,j}$ is the error probability associated with the estimation of $\overline{n}^{1,1}_{X,i,j}$ and $\epsilon_{11x} = \sum_{\{i,j\}=\{u,v,w\}} \epsilon_{11x,i,j}$.

Similar equations can be written for the Z basis, defining with confidence $1 - \epsilon_{11z}$ the bounds

$\overline{n}_Z^{1,1}$ and $\underline{n}_Z^{1,1}$ appearing in Step 5 of the MDI-QKD protocol

$$\overline{n}_Z^{1,1} = s^2 e^{-2s} N_{ZZ} + \sqrt{N_{ZZ}/2 \ln(1/\epsilon_{11z})}, \tag{22}$$

$$\underline{n}_Z^{1,1} = \max\left[s^2 e^{-2s} N_{ZZ} - \sqrt{N_{ZZ}/2 \ln(1/\epsilon_{11z})}, 1\right], \tag{23}$$

with N_{ZZ} counting the total runs where Alice and Bob choose the basis Z. To complete Step 5, we need to estimate the single-photon quantities in the data basis. Because of our assumption of ideal equipment for Alice and Bob, the basis choice is an operation of random sampling in a given population of data available to the users. In a counterfactual protocol, the basis could even be decided after all the pulses have been measured and announced by Charlie. Therefore, if a certain quantity has been measured or estimated in one basis, selected at random, we expect that the same quantity takes on a similar values in the other basis.

We specialise this argument to decoy-state MDI-QKD by writing the following equations, which are analogous to Eqs. (4) and (5):

$$y_Z^{1,1} \approx y_X^{1,1}, \tag{24}$$

$$e_{\mathrm{ph},Z}^{1,1} \approx e_X^{1,1}. \tag{25}$$

The approximate equality signs emphasise that we are considering the finite-size scenario. In order to estimate the Z-basis quantities in Eqs. (24) and (25) from the X-basis ones, we use a bound obtained in Ref. [14] for random sampling without replacement. To simplify the presentation, we use here the same notation as in [14].

Suppose we have a total sample of $n_x + n_z$ events, associated with the X (label x) or the Z (label z) basis in a random way, and that we have measured the single-photon bit error rate in the X basis e_{bx}. Then the quantity $\overline{e}_{pz} = e_{bx} + \theta_x$ represents an upper bound for the single-photon phase error rate in the Z basis, with θ_x a positive parameter to be determined later on. The failure probability associated with this bound is given by the joint probability that Bob measures a bit error rate e_{bx} in the X basis and Eve causes a phase error rate e_{pz} in the Z basis larger than $e_{bx} + \theta_x$. This failure probability can be made arbitrarily small (see Eq. (18) in [14]),

$$P_{\theta_x} = \Pr(e_{pz} \geq e_{bx} + \theta_x, e_{bx}) \tag{26}$$

$$\leq \Pr(e_{pz} \geq e_{bx} + \theta_x | e_{bx}) \tag{27}$$

$$< 2^{-(n_x+n_z)\xi_x(\theta_x)} \sqrt{\frac{n_x + n_z}{e_{bx}(1-e_{bx}) n_x n_z}}, \tag{28}$$

where $\xi_x(\theta_x) = h(e_{bx} + \theta_x - q_x\theta_x) - q_x h(e_{bx}) - (1-q_x) h(e_{bx} + \theta_x)$, h is the binary entropy function and $q_x = n_x/(n_x + n_z)$. By setting P_{θ_x} smaller than a given threshold ϵ_{xx}, we can determine the

value of θ_x and $\bar{e}_{pz}$. Similarly for the Z basis we have

$$P_{\theta_z} = \Pr(e_{px} \geq e_{bz} + \theta_z, e_{bz}) \tag{29}$$

$$< 2^{-(n_x+n_z)\xi_z(\theta_z)} \sqrt{\frac{n_x + n_z}{e_{bz}(1-e_{bz})n_x n_z}}, \tag{30}$$

with $\xi_z(\theta_z) = h(e_{bz} + \theta_z - q_z\theta_z) - q_z h(e_{bz}) - (1-q_z)h(e_{bz} + \theta_z)$ and $q_z = n_z/(n_x + n_z)$. Note that this last expression could be used to estimate either an upper bound for the X-basis phase error rate e_{px} or even a lower bound for the Z-basis bit error rate e_{bz}. In fact we have

$$P_{\theta_z} = \Pr(e_{px}, e_{bz} \leq e_{px} - \theta_z), \tag{31}$$

so we can set $\underline{e}_{bz} = e_{px} - \theta_z$ and be confident that the actual bit error rate in the (supposedly non-measured) Z basis is larger than $\underline{e}_{bz}$, estimated from the (supposedly measured) phase error rate in the X basis. We can now derive sensible bounds for the single-photon quantities in the Z basis of MDI-QKD.

For the single-photon yield, we first note that Eqs. (30), (31) still hold if we replace $y_Z^{1,1} \to e_{bz}$ and $y_X^{1,1} \to e_{px}$, because the yields, as the error rates, are probabilities, i.e. real positive numbers smaller than 1. After this substitution, we obtain

$$\Pr(y_X^{1,1}, y_Z^{1,1} \leq y_X^{1,1} - \theta_z) < 2^{-(\underline{n}_X^{1,1} + \underline{n}_Z^{1,1})\xi_z(\theta_z)} \sqrt{\frac{\underline{n}_X^{1,1} + \underline{n}_Z^{1,1}}{y_Z^{1,1}(1-y_Z^{1,1})\underline{n}_X^{1,1}\underline{n}_Z^{1,1}}}. \tag{32}$$

In Eq. (32), we have replaced the generic populations n_x and n_z by the lower bounds for the single-photon preparations given by Eqs. (21) and (23). This is conservative because minimising the populations always leads to worse bounds in a random sampling process. By bounding the R.H.S. of Eq. (32) with a failure probability $\epsilon_{y_{z|x}}$, we can obtain θ_z and then $\underline{y}_Z^{1,1}$ as expressions of $\epsilon_{y_{z|x}}$. In our experiment, we proceed the other way around. We fix θ_z first, then derive $\underline{y}_Z^{1,1} = \underline{y}_X^{1,1} - \theta_z$ and then calculate the corresponding error probability. We choose θ_z so to have $\epsilon_{y_{z|x}}$ always at least as small as 10^{-25}, an entirely negligible value.

For the phase error rate, we can use directly Eqs. (26)-(28) to obtain

$$\Pr(e_{\text{ph},Z}^{1,1} \geq e_X^{1,1} + \theta_x, e_X^{1,1}) < 2^{-(\underline{S}_X^{1,1} + \underline{S}_Z^{1,1})\xi_x(\theta_x)} \sqrt{\frac{\underline{S}_X^{1,1} + \underline{S}_Z^{1,1}}{e_X^{1,1}(1-e_X^{1,1})\underline{S}_X^{1,1}\underline{S}_Z^{1,1}}}. \tag{33}$$

In the above equation, we have included the lower bounds for the single-photon detection events in the X and Z bases, respectively, from which the error rates are estimated. Explicitly, they can be obtained as $\underline{S}_Z^{1,1} = \lfloor\, \underline{y}_Z^{1,1}\ \underline{n}_Z^{1,1} \rfloor$ and $\underline{S}_X^{1,1} = \lfloor\, \underline{y}_X^{1,1}\ \underline{n}_X^{1,1} \rfloor$. The upper bound for the phase error rate in the Z basis is then given by $\bar{e}_{\text{ph},Z}^{1,1} = \bar{e}_X^{1,1} + \theta_x$, where θ_x is obtained by bounding the R.H.S. of Eq. (33) with a failure probability $\epsilon_{e_{z|x}}$ decided beforehand by the users. Also in this case, we

choose θ_x large enough to make $\epsilon_{e_{z|x}} < 10^{-25}$, a value entirely negligible.

In the main text, we dropped the subscript Z from the quantities in the data basis for readability purposes. The parameters of the protocol are set to obtain composable security [9] with overall security parameter $\lesssim 10^{-10}$, as in [7].

MDI-QKD\EP in reconfigurable MDI/QKD

This protocol aims at distilling quantum digital signatures on the MDI-QKD link between Alice and Bob. It starts from the single-photon bounds $\underline{S}_Z^{1,1}$ and $\overline{e}_{\text{ph},Z}^{1,1}$ derived for the whole Z basis data set using the above MDI-QKD protocol and adjust them to take into account the statistical fluctuations of the block of bits forming the signatures. The specific steps are as follow:

MDI-QKD\EP protocol (signature)

---

1. - 5. Same steps as in the MDI-QKD protocol. From these steps the users estimate the bounds for the single-photon quantities, in particular $\underline{S}_Z^{1,1}$ and $\overline{e}_{\text{ph},Z}^{1,1}$, using the methods described in Sec. .

6. From the total counts in the Z basis the users select a random subset of $C_{\text{test}}^{s,s}$ bits and measure the QBER $E_{\text{test}}^{s,s}$ in this subset.

7. The remaining counts in the Z basis are randomly grouped into M_{sig} subsets, each of size $C_{\text{sig}}^{s,s}$. From the concatenation of the bits in each subset, the users will form M_{sig} digital signatures.

8. The users estimate an upper bound for the QBER in the signatures subsets, $\overline{E}_{\text{sig}}^{s,s}$ using the measured value of $E_{\text{test}}^{s,s}$ and Hoeffding's inequality, through Eq. (3) in the main text.

9. The users estimate the bounds for the single-photon quantities in the signatures subsets, in particular $\underline{S}_{\text{sig}}^{1,1}$ and $\overline{e}_{\text{ph,sig}}^{1,1}$, using Eqs. (35) and (36), respectively, reported below.

10. The users estimate all the parameters relevant to the quantum digital signatures from the equations given below and the procedure described in the main text.

For consistency, we here rewrite Eq. (3) of the main text, mentioned in Step 8 of the above protocol, which provides the worst-case estimate of the QBER in each signature block, $\overline{E}_{\text{sig}}^{s,s}$

$$\overline{E}_{\text{sig}}^{s,s} = E_{\text{test}}^{s,s} + \frac{1}{C_{\text{sig}}^{s,s}} \sqrt{\frac{(C_{\text{sig}}^{s,s}+1)(C_{\text{sig}}^{s,s}+C_{\text{test}}^{s,s})}{2\, C_{\text{test}}^{s,s}} \ln(1/\epsilon_H)}. \tag{34}$$

Similar to the QKD protocol described above, this equation is derived by applying Serfling's inequality [8] to the data sample formed by $C_{\text{test}}^{s,s} + C_{\text{sig}}^{s,s}$, in which $E_{\text{test}}^{s,s}$ is known because it is directly

measured. The inequality in Eq. (34) entails an overall failure probability ϵ_H, whose value is set equal to 2×10^{-11}.

Eq. (35) mentioned in Step 9 of the above protocol aims at estimating the amount of single-photon detections in a signature subset. From Steps 1-5 of the protocol, the users get the minimum number of single-photon detections $\underline{S}_Z^{1,1}$ in the Z basis data sample, whose size has already been defined as $C_Z^{s,s}$. Therefore, because the $C_{\rm sig}^{s,s}$ bits of the signature subset are randomly selected from the Z basis sample, we can apply a simple proportionality rule and Serfling's inequality [8] to obtain

$$\underline{S}_{\rm sig}^{1,1} = \underline{S}_Z^{1,1} \times \frac{C_{\rm sig}^{s,s}}{C_Z^{s,s}} - \sqrt{\frac{(C_Z^{s,s} - C_{\rm sig}^{s,s})(C_{\rm sig}^{s,s}+1)}{2C_Z^{s,s}} \ln(1/\epsilon_H)}. \tag{35}$$

By the same argument, we can apply Serfling's inequality again to estimate the upper bound for the phase-error rate in the signature population, $\overline{e}_{\rm ph,sig}^{1,1}$,

$$\overline{e}_{\rm ph,sig}^{1,1} = \overline{e}_{{\rm ph},Z}^{1,1} + \frac{1}{\underline{S}_{\rm sig}^{1,1}} \sqrt{\frac{(\underline{S}_Z^{1,1} - \underline{S}_{\rm sig}^{1,1})(\underline{S}_{\rm sig}^{1,1}+1)}{2\ \underline{S}_Z^{1,1}} \ln(1/\epsilon_H)}. \tag{36}$$

Eqs. (35) and (36) contribute to the overall failure probability of the signature distillation protocol by a factor ϵ_H each. By adding these two failure probabilities, the one related to Eq. (34) and the one related to the decoy-state parameter estimation, $\epsilon_{\rm sec}$, we obtain a total failure probability less than 10^{-10}.

From the quantities specified above, the users can estimate the probability $p_E^{\rm MDI}$ mentioned in the main text by solving the equation

$$h\left(p_E^{\rm MDI}\right) = \frac{\underline{S}_{\rm sig}^{1,1}}{C_{\rm sig}^{s,s}}[1 - h(\overline{e}_{\rm ph,sig}^{1,1})]. \tag{37}$$

Using an analysis similar similar to that in the previous Section , we choose the values of s_{ab} and s_{vb} such that $\overline{E}_{\rm sig}^{s,s} < s_{ab} < s_{vb} < p_E^{\rm MDI}$. We obtain the signature length $L_{\rm sig}^{\rm MDI}$ by inverting the relation

$$P_{\rm rep}^{\rm MDI} \leq \exp\left[-(s_{vb} - s_{ab})^2 L_{\rm sig}^{\rm MDI}/4\right] \leq 0.5 \times 10^{-10}. \tag{38}$$

The probability of honest abort is the same as defined in Eq. (14), and for the probability of forging we use Eq.(15) by replacing the parameters, $L_{\rm sig}^{\rm QKD}$, $\underline{S}_{\rm sig}^{1}$ and $\overline{e}_{\rm ph,sig}^{1}$ respectively with $L_{\rm sig}^{\rm MDI}$, $\underline{S}_{\rm sig}^{1,1}$ and $\overline{e}_{\rm ph,sig}^{1,1}$. The overall probability of repudiation for the signature protocol is the sum of $P_{\rm rep}^{\rm MDI}$ and $P_{\rm rep}^{\rm QKD}$. That is,

$$P_{\rm rep} = P_{\rm rep}^{\rm QKD} + P_{\rm rep}^{\rm MDI} \leq 10^{-10}. \tag{39}$$

QDS in reconfigurable MDI/QKD

We outline the QDS protocol for three parties, with a signer, Alice, and two recipients, Bob and Charlie, as depicted in Fig. 4 of the main text. We assume that between Alice and Bob, and between Alice and Charlie, there exist authenticated classical channels. There is no need for a "direct" fibre link between Alice and Bob, but using Charlie as an untrusted intermediate node, they are linked using MDI-QKD without error correction and privacy amplification (denoted by "MDI-QKD\EP"); see Section for more details. This results in correlated bit strings held by Alice and Bob. In a similar way, Alice and Charlie uses the QKD\EP protocol to generate correlated bit strings, with more details in Section . Bob and Charlie share a QKD link (more details in Section), which can be used to transmit classical messages in full secrecy.

The QDS scheme has two stages, a distribution stage, where all quantum communication takes place, and a messaging stage, which can occur much later, and where only classical communication is used. We will describe the procedure for signing a one-bit message. For signing longer messages, the procedure can be suitably iterated.

Distribution stage

(1) For each possible future message $i = 0$ or 1, Alice uses MDI-QKD\EP to generate two different correlated bit strings, A_0^B and A_1^B, with Bob. The superscript denotes the participant with whom Alice performed the key generation protocol, and the subscript represents the future message, which is to be decided later by her. Each string is of length $C_{\text{sig}}^{s,s}$. Also, she uses QKD\EP to generate another two different correlated bit strings A_0^C and A_1^C with Charlie, each one of length C_{sig}^{s}. Bob holds the strings K_0^B, K_1^B and Charlie holds the strings K_0^C, K_1^C; these are correlated with Alice's corresponding strings. Because of the key generation protocols (MDI-QKD\EP and QKD\EP), it will be guaranteed that A_0^B contains fewer mismatches with K_0^B than does any string produced by an eavesdropper, and similarly for the other pairs of strings. Alice's signature for the future message i will be $\text{SIG}_i = (A_i^B, A_i^C)$. The fact that only Alice knows both signature strings for a message i protects the protocol against forging.

(2) For each possible future message, Bob and Charlie symmetrize their keys. This is done by each of them randomly choosing half of the bit values in their keys (K_i^B, K_i^C) and sending these bit values (as well as the corresponding positions) to the other participant using their secret classical channel, here implemented using a separate full QKD system and the protocol in Sec. . This will ensure that Alice cannot make Bob and Charlie disagree on the validity of a signature, if a message is forwarded from Bob to Charlie or vice versa in the messaging stage. If Bob (or Charlie) chooses to forward an element of K_i^B (or K_i^C) in the distribution stage to Charlie (or Bob), he will not, if he is honest, further use it to check the validity of a signature. Bob and Charlie will only use the bits they did not forward, and those received from the other participant. This is not strictly necessary, but simplifies security proofs as it makes the situation more symmetric from Alice's point of view.

We denote the symmetrized keys by S_i^B and S_i^C, with the superscript indicating whether the key is held by Bob or Charlie. Bob (and Charlie) keep a record of whether an element in S_i^B (S_i^C) came directly from Alice or whether it was forwarded to him by Charlie (or Bob).

At the end of the distribution stage, for each future message, Bob holds only partial information of Charlie's key (K_i^C) and this protects the protocol against forging by Bob (and similarly against forging by Charlie). Alice has no information on whether it is Bob's S_i^B or Charlie's S_i^C that contains a particular element of the string (K_i^B, K_i^C) and this protects against repudiation. That is, Alice cannot send a message that will be accepted by one participant and rejected by the other.

Messaging stage

(1) To send a signed one-bit message i, Alice sends (i, SIG_i) to the desired recipient (say Bob).

(2) Bob checks whether (i, SIG_i) matches his S_i^B, and records the number of mismatches he finds. He separately checks the part of his key received directly from Alice and the part of the key received from Charlie. If there are fewer than $C_{\mathrm{sig}}^{s,s} s_{ab}/2$ mismatches in the half of the key coming from Alice and fewer than $(C_{\mathrm{sig}}^{s,s} s_{ab} + C_{\mathrm{sig}}^{s} s_{ac})/4$ mismatches in the half of the key coming from Charlie, Bob accepts the message. Here s_{ab} and s_{ac} are small thresholds that are smaller than $1/2$. These thresholds are determined by the observed experimental parameters and the desired security level of the protocol.

(3) To forward the message to Charlie, Bob forwards the pair (i, SIG_i) that he received from Alice.

(4) Charlie tests for mismatches in a similar way, but using a different threshold in order to protect against repudiation by Alice. He accepts the forwarded message if the number of mismatches in the half received by Alice is below $C_{\mathrm{sig}}^{s} s_{vc}/2$ and those in the half received by Bob are below $(C_{\mathrm{sig}}^{s,s} s_{vb} + C_{\mathrm{sig}}^{s} s_{vc})/4$, where s_{vc} and s_{vb} are another thresholds, with $0 < s_{ab} < s_{vb} < 1/2$ and $0 < s_{ac} < s_{vc} < 1/2$.

Supplementary References

[1] Bennett, C. H. & Brassard, G. in *Proc. IEEE Int. Conf. on Computers, Systems and Signal Processing* 175-179 (1984).

[2] Beaudry, N. J., Moroder, T. & Lütkenhaus, N. Squashing models for optical measurements in quantum communication. *Phys. Rev. Lett.* **101**, 093601 (2008).

[3] Tsurumaru, T. & Tamaki, K. Security proof for quantum-key-distribution systems with threshold detectors. *Phys. Rev. A* **78**, 032302 (2008).

[4] Fung, C.-H. F., Chau, H. F. & Lo, H.-K. Universal squash model for optical communications using linear optics and threshold detectors. *Phys. Rev. A* **84**, 020303(R) (2011).

[5] Lim, C. C. W., Curty, M., Walenta, N., Xu, F. & Zbinden, H. Concise security bounds for practical decoy-state quantum key distribution. *Phys. Rev. A* **89**, 022307 (2014).

[6] Hoeffding, W. Probability inequalities for sums of bounded random variables. *J. Amer. Statist. Assoc.* **58**, 13-30 (1963).

[7] L. C. Comandar *et al.*, Quantum cryptography without detector vulnerabilities using optically-seeded lasers. *Nature Photon.* **10**, 312-315 (2016).

[8] Serfling, R. J. Probability inequalities for the sum in sampling without replacement. *Ann. Statist.* **2**, 39-48 (1974).

[9] Curty, M. *et al.*, Finite-key analysis for measurement-device-independent quantum key distribution. *Nature Comm.* **5**, 3732 (2014).

[10] Amiri, R., Wallden, P., Kent, A. & Andersson, E. Secure quantum signatures using insecure quantum channels. *Phys. Rev. A* **93**, 032325 (2016).

[11] Puthoor, I. V., Amiri, R., Wallden, P., Curty, M. & Andersson, E. Measurement-device-independent quantum digital signatures. *Phys. Rev. A* **94**, 022328 (2016).

[12] Lo, H.-K., Curty, M. & Qi, B. Measurement-Device-Independent Quantum Key Distribution. *Phys. Rev. Lett.* **108**, 130503 (2012).

[13] Zhou, Y.-H., Yu, Z.-W. & Wang, X.-B. Making the decoy-state measurement-device-independent quantum key distribution practically useful. *Phys. Rev. A* **93**, 042324 (2016).

[14] Fung, C.-H. F., Ma, X. & Chau H. F. Practical issues in quantum-key-distribution postprocessing. *Phys. Rev. A* **81**, 012318 (2010).
